# Antiviral Interferon-Beta Signaling Induced by Designed Transcription Activator-Like Effectors (TALE)

**DOI:** 10.1371/journal.pone.0114288

**Published:** 2014-12-03

**Authors:** Ivonne Renner, Nancy Funk, Rene Geissler, Susann Friedrich, Anika Penzel, Sven-Erik Behrens

**Affiliations:** Institute of Biochemistry and Biotechnology, Section Microbial Biotechnology, Martin Luther University Halle-Wittenberg, Faculty of Life Sciences (NFI), Kurt-Mothes-Str. 3, D-06120, Halle/Saale, Germany; Thomas Jefferson University, United States of America

## Abstract

Here we show that designed transcription activator-like effectors (TALEs) that bind to defined areas of the interferon beta promoter are capable to induce IFN-beta expression and signaling in human cells. Importantly, TALE-mediated IFN-beta signaling occurs independently of pathogen pattern recognition but effectively prohibits viral RNA replication as demonstrated with a hepatitis C virus replicon. TALEs were thus indicated to be valuable tools in various applications addressing, for example, virus-host interactions.

## Introduction

TALEs were originally characterized as virulence factors of plant pathogenic bacteria that reprogram gene transcription of the host cells. TALEs contain a DNA binding domain that is composed of similar tandem repeats of typically 34 amino acids. For transcription activation, each repeat binds one base pair of the target DNA, and a repeat-variable di-residue (RVD) specifies the bound base [1]–[4]. Thus, ‘designer TALEs’ containing a defined order of repeats and a suitable transcription activation domain can be constructed and applied to induce the transcription of human genes [5]–[7].

**Figure 1:**
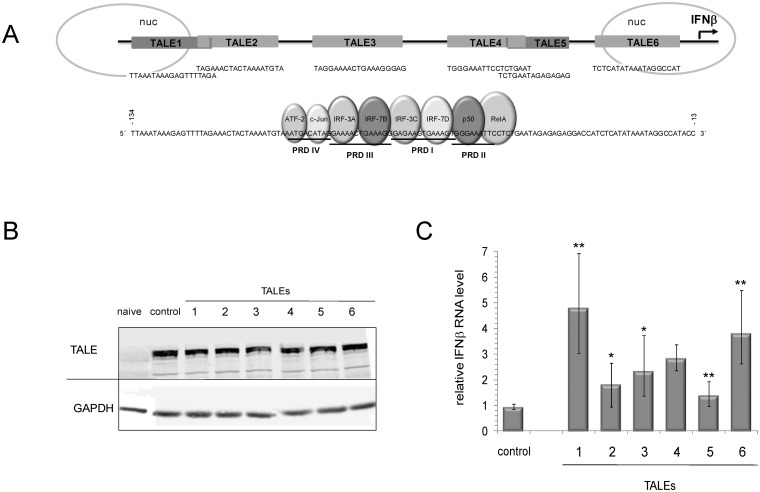
TALE-induced expression of IFN-beta. *(A)* Organization of the IFN-beta promoter (region at −134 to −13 nt upstream of transcriptional start). The nucleosome (nuc) and TALE binding sites are schematized as well as the interaction sites of transcription factors that associate with the IFN-beta promoter in activated cells forming the enhanceosome. *(B)* Expression levels of TALEs1–6 and of a control TALE (without human target sequence) in Huh7 cells, detected by western blot in comparison with the housekeeping protein Glyceraldehyde-3-Phosphate Dehydrogenase (GAPDH). *(C)* IFN-beta mRNA level in Huh7 cells at 24 h post-transfection (p.t.) of TALE-expressing plasmids (IFN-beta mRNA level in control set as 1). Error bars indicate standard deviations of seven independent experiments. (* = *P*<0,05, ** = *P*<0,01).

An attractive target for transcription activation is the cytokine IFN-beta, which is well characterized regarding its antiviral activity and also used during treatment of multiple sclerosis [8], [9]. The IFN-beta promoter mainly consists of an enhancer that is flanked by two nucleosomes, one masking the transcriptional TATA-box (**Fig. 1A**).

The transcription of the IFN-beta gene is stringently regulated. During cell activation, which may be provoked by interactions of cellular pattern recognition receptors (PRR) with pathogen associated molecular pattern (PAMP), signaling cascades induce the assembly of transcription and nucleosome remodeling factors (the so-called ‘enhanceosome’) at the enhancer’s positive regulatory domains (PRD; **Fig. 1A**). This, in turn, enables the association of the TATA-binding protein and Pol II-mediated transcription to initiate [10]–[13]. Accordingly, in non-activated cells (i.e., in the absence of the enhanceosome), the IFN-beta promoter was considered to be accessible for the binding and transcription-inducing activity of TALEs.

In this report we show that TALEs directed to bind to certain sites of the IFN-beta promoter induce an effective antiviral signaling cascade also in the absence of an external stimulus.

## Methods

### Cloning of TALE genes

Using a repeat library and the Golden TALE technology [5] six single-repeat modules were ligated into assembly vectors as a six-repeat array by cut-ligation using *Bpi*I. The procedure was essentially performed as described by Geissler et al. [5]. To assemble the complete TALE-coding sequence, three six-repeat arrays were ligated with modules encoding a green fluorescent protein (GFP) tag, the N- and C-terminus, and the herpes simplex virus VP16 transcription activation domain (C-terminal 68 aa), respectively, into a pcDNA3 (Invitrogen) derivative using *Bsa*I. The amino acid sequence of the applied TALEs is given in **Table S1** in File S1.

### Construction of reporter plasmid

The plasmid was based on pF12A RM Flexi (Promega) where the barnase gene was replaced by a luciferase gene. To generate plasmids containing the TALE recognition sites, pF12A RM (Luc) was amplified with primers containing target boxes. The promoter and 5′ untranslated region (675 bp) of the *IFN-beta1* promoter were amplified by PCR and inserted into the luciferase reporter plasmid [5].

### *In* *vitro* transcription

The replicon-encoding plasmid pSGR-JFH1 was kindly provided by Dr. Wakita (Tokyo Metropolitan Institute for Neuroscience) and modified as described in [14]. The plasmid was digested with XbaI and transcribed by run-off *in vitro* transcription (standard protocol) with T7 RNA-polymerase (Stratagene) using the protocol of Geissler et al. 2012 [14].

### Cell culturing and transfection conditions

Huh7 cells [14] were cultured in DMEM (Invitrogen) supplemented with 10% FCS (PAN-Biotech), 1% penicillin/streptomycin (Invitrogen), 0.1% d-Biotin and 0.1% hypoxanthin (Sigma) [15]. Transfection of plasmids was performed with 70% confluent cells, 20 µg plasmid DNA/10 ml growth medium using Turbofect (Fermentas) and the manufacturer’s instructions. The replicon RNA was transfected using 300 ng (ca. 100 fmol) and the Bio-Rad Gene Pulser II (1 pulse without controller at 0.2 kV and 950 µF).

### Western blot

The western-blot analysis was performed at 24 h p.t. of the TALE-expressing plasmids. Ca. 2×10^6^ cells were centrifuged at 1000×*g*, washed with phosphate buffer saline and lysed by 1x lysis buffer (Promega). The protein amount was determined by a standard Bradford assay (BioRad) and 20 µg of total cell protein separated per lane by SDS-PAGE. Following the transfer to nitrocellulose (Millipore), the reaction was carried out using standard conditions and the following antibodies: anti-GFP (Invitrogen A-6455) 1∶2000, anti-GAPDH (Santa Cruz sc-47724) 1∶15000; secondary antibodies (each at 1∶5000 dilution) anti-rabbit (Licor Cw 800 926-32213), anti-mouse (Licor Cw800 926-32212).

### RT-PCR analysis

For qRT-PCR analysis, RNA was isolated from ca. 2×10^6^ cells with Trizol at the indicated time points and the qRT-PCR performed using Revert Aid reverse transcriptase (Thermo) and the PCR-MasterMix qRT (Roboklon GmbH, Germany). For reverse transcription of total mRNA, an oligodT_19_ primer was applied; for reverse transcription of the HCV replicon JFH, we applied the primer JFH reverse (ACA TGA TCT GCA GAG AGA CCA G). The RT conditions were 1 h at 42°C (amounts of applied RNA 500 ng); the PCR conditions were 94°C, 15 sec; 60°C, 25 sec; 72°C 25 sec×40 cycles. The HCV RNA levels were normalized to GAPDH RNA as internal control. For further details, see Geissler et al. [14]. All applied DNA oligonucleotides (purchased from Eurofins, Germany) are summarized in **Table S2** in File S1.

### Data evaluation and statistics

Data evaluation and statistics were done as described previously [14].

### Ethics statement

Not applicable.

## Results and Discussion

Following earlier work, which revealed the general option to induce IFN-beta expression in human cells by a TALE [5], this study aimed at investigating if an entire antiviral IFN-beta signaling cascade may be navigated by designed effectors. For this purpose, we generated a set of six TALE-expressing constructs applying the ‘Golden TALE technology’ [5]. The corresponding effectors were designed to bind to different regions of the IFN-beta promoter (see **Fig. 1A** and **Table 1**).

**Table 1:** TALE binding sites within the IFN-beta promoter (see also Fig. 1A).

TALE	target sequence
control	T CGG TCT GGC TTG ACA TGA
1	T TAA ATA AAG AGT TTT AGA
2	T AGA AAC TAC TAA AAT GTA
3	T AGG AAA ACT GAA AGG GAG
4	T GGG AAA TTC CTC TGA AT
5	T CTG AAT AGA GAG AG
6	T CTC ATA TAA ATA GGC CAT

After transient transfection of the expression plasmids, all six effectors were comparably expressed in Huh7 cells (human hepatoma cells). This was demonstrated by western-blot that detected the TALEs *via* a fused green fluorescent protein (GFP) reporter (**Fig. 1B**). In a subsequent experiment, we expressed the individual TALEs in Huh7 cells and measured the amount of IFN-beta mRNA by qRT-PCR (**Fig. 1C**). This data revealed that with promoter-associating TALEs, the transcription of the IFN-beta gene was up to 4-fold increased in comparison to experiments with a non-related TALE. Interestingly, most effective were TALEs1 and 6 that were binding to the sites of the promoter that were indicated to be covered by nucleosomes (**Fig. 1A and C**). This suggests that the effectors, besides attracting the Pol II transcription machinery, may also facilitate nucleosomal remodeling.

**Figure 2:**
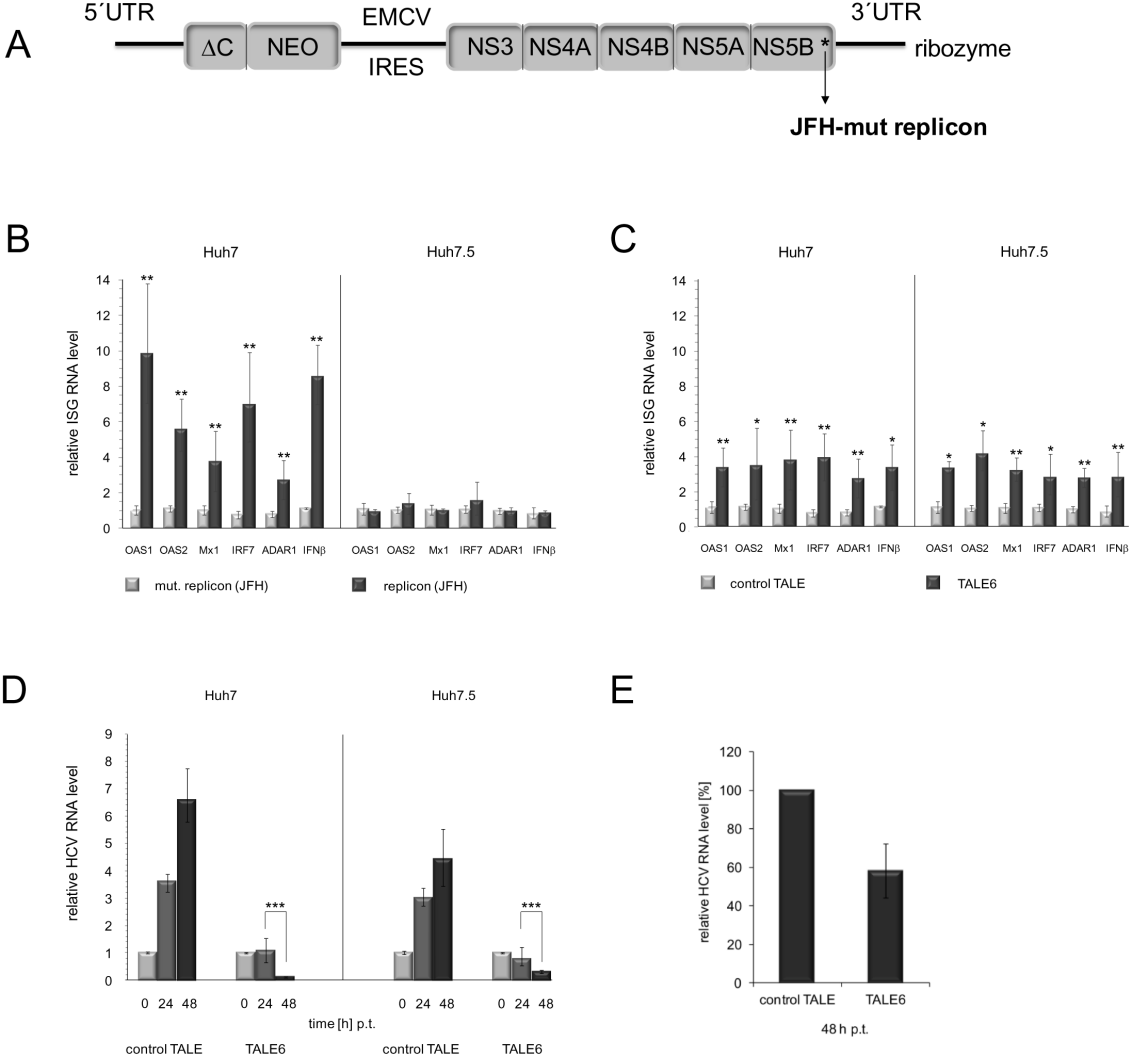
TALE-induced inhibition of HCV RNA replication. *(A)* Genomic organization of the applied HCV replicon [25]. The untranslated regions (UTRs) and the Encephalomyocarditis virus internal ribosomal entry site (EMCV IRES) are depicted as lines, the regions encoding a part of the core protein (ΔC) the neomycine resistance gene and the viral proteins NS3 to NS5B are depicted as boxes. A star in the NS5B coding region indicates that with the non-replicative replicon, this protein was mutated [28]. The replicon RNA was expressed with a 3′-ribozyme, which improved the generation of the authentic viral RNA’s 3′-end [28]. *(B)* Induction of IFN-beta and ISGs in Huh7 and Huh7.5 cells that were transfected with the TALE6-encoding plasmid (mRNA level measured with the control TALE in Huh7 and Huh7.5 cells set as 1). The relative mRNA levels of IFN-beta and of the indicated ISGs were determined by qRT-PCR at 24 h p.t. (mRNA level measured with the non-replicative replicon in Huh7 and Huh7.5 set as 1). *(C)* Analogous experiment performed with cells transfected with a mutant (non-replicative) or replicative HCV replicon. *(D)* Replication efficiency of HCV replicon in Huh7 and Huh7.5 cells transfected with either TALE6 or control TALE. The relative viral RNA levels were measured at the indicated time points (HCV RNA levels measured at time 0 p.t. set as 1). Error bars indicate standard deviations of five independent experiments (* = *P*<0,05, ** = *P*<0,01, *** = *P*<0,001; set significance level α = 0,05). *(E)* Huh7 cells were transfected with HCV replicon RNA. After 6 h, i.e., a time frame where significant HCV replication is detectable by qRT-PCR [14], the cells were transfected with plasmids expressing the control TALE or TALE6. At 48 h post transfection (pt) of the replicon RNA, the relative viral RNA levels were measured (HCV RNA-levels of the control set to 100%). Error bars indicate standard deviations of three independent experiments.

Secreted IFNs function by binding to the IFN receptor (IFNAR) of neighboring cells and by activating the canonical JAK/STAT pathway. This leads to the formation of interferon-stimulated gene factor complexes (ISGF3) that drive the transcription of interferon-stimulated genes (ISGs). ISGs encode antiviral proteins like OAS (2′-5′-oligoadenylate synthetase), MX (GTPase), ADAR (adenosine deaminase) and signaling proteins as the interferon regulatory factor IRF7 [16]–[18]. To understand next if IFN-beta expression correlated with ISG expression, we performed qRT-PCR that measured the mRNA levels of IFN-beta side-by-side with those of OAS1 and 2, MX1, IRF7 and ADAR1. This was done with two cell types, Huh7 and Huh7.5. Huh7.5 differ from Huh7 such that RIG-I (retinoic acid-inducible gene I), an important intracellular PRR [19] is defective in these cells [20], [21]. Thus, with the pathogen hepatitis C virus (HCV) it is well understood that PAMP as the tri-phosphate at the viral RNA’s 5′-end and an HCV-specific RNA motif in the 3′ untranslated region (3′UTR) of the viral genome are recognized by RIG-I and that RIG-I-induced signaling cascades lead to the activation of transcription factors that regulate IFN gene expression [22]–[25]. To compare HCV- and TALE-induced IFN-beta signalling, both cell types were transfected either with an HCV subgenomic RNA replicon [26] (**Fig. 2A**) or with the TALE6-expressing plasmid. Interestingly, as shown in **Fig. 2C**, TALE6 induced the expression of IFN-beta and ISGs in both cell types, while replicating HCV RNA did so only in Huh7 (**Fig. 2B**). Controls performed with a non-replicating HCV RNA or a non-specific TALE showed neither an induction of IFN-beta nor of ISGs (**Fig. 2B and C**). This data highlights that TALE6 triggered the entire IFN-beta signaling cascade. Most importantly, with the experiment applying Huh7.5 cells, we confirmed that TALE-induced IFN-beta signaling occurred independently of PAMP signaling.

In a final experiment, we addressed the question if the TALE6-mediated induction of IFN-beta and ISGs was capable to interfere with viral RNA replication. For this, Huh7 and Huh7.5 cells were transfected with the TALE6-encoding plasmid and, 24 h later, with the HCV replicon. As shown in **Fig. 2D**, prior expression of TALE6 effectively inhibited viral RNA replication while expression of a non-related TALE did not. A considerable inhibitory effect on viral replication was also observed in cells when we expressed TALE6 in Huh7 cells where HCV replication was already established (**Fig. 2E**).

The activation of IFN-beta gene expression *via* PRR-PAMP signaling is a well-studied model of how transcriptional output is regulated in the cell. Here we demonstrate that TALEs, by operating directly on the transcriptional level, bypass PAMP/RIG-I-mediated signaling. Moreover, TALE-triggered IFN-beta signaling effectively prohibits HCV replication in hepatoma cells. Thus, in comparison to the IFN-beta response that is induced by the HCV replicon in Huh-7 cells (**Fig. 2B**), the TALE6-stimulated IFN-beta and ISG expression (**Fig. 2C**) leads to a clearly detectable decrease in the level of viral RNA (**Fig. 2D**). We explained this by the multiple ways of how viral factors, which are also encoded by the applied replicon may inhibit IFN signaling [27]. For example, the HCV NS3/4A protease inhibits HCV PAMP-RIG-I signaling by proteolytic degradation of a RIG-I signaling adapter [28].

Taken together, our data recommend IFN-beta inducing TALEs as potentially valuable tools for future vaccination or treatment applications. Specifically designed TALEs may also be helpful to unravel the function of yet insufficiently characterized host factors participating in (antiviral) cell signaling or viral replication.

## Supporting Information

File S1 **Supporting tables.** Table S1, Organization and amino acid sequences of applied TALEs. Table S2, DNA oligonucleotides applied for qRT-PCR.
(DOCX)

## References

[1] Boch J, Scholze H, Schornack S, Landgraf A, Hahn S, et al (2009) Breaking the code of DNA binding specificity of TAL-Type III effectors. Science 326:1509–1512. 19933107 10.1126/science.1178811

[2] Moscou MJ, Bogdanove AJ (2009) A simple cipher governs DNA recognition by TAL effectors. Science 326:1501. 19933106 10.1126/science.1178817

[3] Boch J, Bonas U (2010) *Xanthomonas* AvrBs3 family-type III effectors: discovery and function. Annu Rev Phytopathol 48:419–436. 19400638 10.1146/annurev-phyto-080508-081936

[4] Doyle EL, Stoddard BL, Voytas DF, Bogdanove AJ (2013) TAL effectors: highly adaptable phytobacterial virulence factors and readily engineered DNA-targeting proteins. Trends Cell Biol 23:390–398. 23707478 10.1016/j.tcb.2013.04.003 PMC3729746

[5] Geißler R, Scholze H, Hahn S, Streubel J, Bonas U, et al (2011) Transcriptional activators of human genes with programmable DNA. PLoS ONE 6:e19509. 21625585 10.1371/journal.pone.0019509 PMC3098229

[6] Zhang F, Cong L, Lodato S, Kosuri S, Church GM, et al (2011) Efficient construction of sequence-specific TAL effectors for modulating mammalian transcription. Nat Biotechnol 29:149–153. 21248753 10.1038/nbt.1775 PMC3084533

[7] Miller JC, Tan S, Qiao G, Barlow KA, Wang J, et al (2011) A TALE nuclease architecture for efficient genome editing. Nat. Biotechnol. 29:143–148. 10.1038/nbt.1755 21179091

[8] Stetson DB, Medzhitov R (2006) Type I interferons in host defense. Immunity 25:373–381. 16979569 10.1016/j.immuni.2006.08.007

[9] Trinchieri G (2010) Type I interferon: friend or foe? J Exp Med 207:2053–2063. 20837696 10.1084/jem.20101664 PMC2947062

[10] Agalioti T, Lomvardas S, Parekh B, Yie J, Maniatis T, et al (2000) Ordered recruitment of chromatin modifying and general transcription factors to the IFN-beta promoter. Cell 103:667–678. 11106736 10.1016/s0092-8674(00)00169-0

[11] Panne D, Maniatis T, Harrison SC (2007) An atomic model of the Interferon-beta enhanceosome. Cell 129:1111–1123. 17574024 10.1016/j.cell.2007.05.019 PMC2020837

[12] Ford E, Thanos D (2010) The transcriptional code of human IFN-beta gene expression. Biochim Biophys Acta. 1799:328–336. 20116463 10.1016/j.bbagrm.2010.01.010

[13] Levy DE, Marie IJ, Durbin JE (2011) Induction and function of type I and III interferon in response to viral infection. Curr Opin Virol 1:476–486. 22323926 10.1016/j.coviro.2011.11.001 PMC3272644

[14] Geissler R, Golbik RP, Behrens SE (2012) The DEAD-box helicase DDX3 supports the assembly of functional 80 S ribosomes. Nucleic Acids Res 40:4998–5011. 22323517 10.1093/nar/gks070 PMC3367175

[15] Isken O, Baroth M, Grassmann CW, Weinlich S, Ostareck DH, et al (2007) Nuclear factors are involved in hepatitis C virus RNA replication. RNA 13:1675–1692. 17684232 10.1261/rna.594207 PMC1986813

[16] Platanias LC (2005) Mechanisms of type I and type II interferon mediated signalling. Nat Rev Immunol 5:375–386. 15864272 10.1038/nri1604

[17] MacMicking JD (2012) Interferon-inducible effector mechanisms in cell-autonomous immunity. Nat Rev Immunol 12:367–382. 22531325 10.1038/nri3210 PMC4150610

[18] Schoggins JW, Wilson SJ, Panis M, Murphy MY, Jones CT, et al (2011) A diverse range of gene products are effectors of the type I interferon antiviral response. Nature 472:481–487. 21478870 10.1038/nature09907 PMC3409588

[19] Yoneyama M, Kikuchi M, Natsukawa T, Shinobu N, Imaizumi T, et al (2004) The RNA helicase RIG-I has an essential function in double-stranded RNA-induced innate antiviral responses. Nat Immunol 5:730–737. 15208624 10.1038/ni1087

[20] Blight KJ, McKeating JA, Rice CM (2002) Highly permissive cell lines for subgenomic and genomic hepatitis C virus RNA replication. J Virol 76:13001–13014. 12438626 10.1128/JVI.76.24.13001-13014.2002 PMC136668

[21] Sumpter R, Loo YM, Foy E, Li K, Yoneyama M, et al (2005) Regulating intracellular antiviral defense and permissiveness to hepatitis C virus RNA replication through a cellular RNA helicase, RIG-I. J Virol 79:2689–2699. 15708988 10.1128/JVI.79.5.2689-2699.2005 PMC548482

[22] Schlee M, Hartmann E, Coch C, Wimmauer V, Janke M, et al (2009) Approaching the RNA ligand for RIG-I? Immunol Rev 227:66–74. 19120476 10.1111/j.1600-065X.2008.00724.x

[23] Saito T, Owen DM, Jiang F, Marcotrigiano J, Gale M (2008) Innate immunity induced by composition-dependent RIG-I recognition of hepatitis C virus RNA. Nature 454:523–527. 18548002 10.1038/nature07106 PMC2856441

[24] Uzri D, Gehrke L (2009) Nucleotide sequences and modifications that determine RIG-I/RNA Binding and signaling activities. J Virol 83:4174–4184. 19224987 10.1128/JVI.02449-08 PMC2668486

[25] Horner S, Gale M (2009) Intracellular innate immune cascades and interferon defenses that control hepatitis C virus. J Interferon Cytokine Res 29:489–498. 19708811 10.1089/jir.2009.0063 PMC2956657

[26] Wakita T, Pietschmann T, Kato T, Date T, Miyamoto M, et al (2005) Production of infectious hepatitis C virus in tissue culture from a cloned viral genome. Nat Med 11:791–905. 15951748 10.1038/nm1268 PMC2918402

[27] Li K, Lemon SM (2013) Innate immune responses in hepatitis C virus infection. Semin Immunopathol 35:53–72. 22868377 10.1007/s00281-012-0332-x PMC3732459

[28] Meylan E, Curran J, Hofmann K, Moradpour D, Binder M, et al (2005) Cardif is an adaptor protein in the RIG-I antiviral pathway and is targeted by hepatitis C virus. Nature 437:1167–1172. 16177806 10.1038/nature04193

