# Supplementary material for: Antiviral Interferon-Beta Signaling Induced by Designed Transcription Activator-Like Effectors (TALE)
Source: PLoS One. 2014 Dec 3;9(12):e114288. doi: 10.1371/journal.pone.0114288 (PMC4255017; doi:10.1371/journal.pone.0114288)
Supplement: File S1 — Supporting tables. Table S1, Organization and amino acid sequences of applied TALEs. Table S2, DNA oligonucleotides applied for qRT-PCR. (DOCX) [file pone.0114288.s001.docx]

**Supporting Information**

**Supporting tables**

**Table S1. Organization and amino acid sequences of applied TALEs.**

Organization:

GFP-tag ͏ N-terminus ͏ repeat domain ͏ C-terminus ͏ VP16 activation domain

Amino acid sequences:

1. control TALE

**KGEELFTGVVPILVELDGDVNGHKFSVSGEGEGDATYGKLTLKFICTTGKLPVPWPTLVTTFTYGVQCFSRYPDHMKQHDFFKSAMPEGYVQERTIFFKDDGNYKTRAEVKFEGDTLVNRIELKGIDFKEDGNILGHKLEYNYNSHNVYIMADKQKNGIKVNFKIRHNIEDGSVQLADHYQQNTPIGDGPVLLPDNHYLSTQSALSKDPNEKRDHMVLLEFVTAAGITHGMDELY͏MDPIRSRTPSPARELLSGPQPDGVQPTADRGVSPPAGGPLDGLPARRTMSRTRLPSPPAPSPAFSADSFSDLLRQFDPSLFNTSLFDSLPPFGAHHTEAATGEWDEVQSGLRAADAPPPTMRVAVTAARPPRAKPAPRRRAAQPSDASPAAQVDLRTLGYSQQQQEKIKPKVRSTVAQHHEALVGHGFTHAHIVALSQHPAALGTVAVKYQDMIAALPEATHEAIVGVGKQWSGARALEALLTVAGELRGPPLQLDTGQLLKIANGGGVTAVEAVHAWRNALTGAP͏**NLTPEQVVAIASHDGGKQALETVQRLLPVLCQAHGLTPEQVVAIASNNGGKQALETVQRLLPVLCQAHGLTPEQVVAIASNNGGKQALETVQRLLPVLCQAHGLTPEQVVAIASNGGGKQALETVQRLLPVLCQAHGLTPEQVVAIASHDGGKQALETVQRLLPVLCQAHGLTPEQVVAIASNGGGKQALETVQRLLPVLCQAHGLTPEQVVAIASNNGGKQALETVQRLLPVLCQAHGLTPEQVVAIASNNGGKQALETVQRLLPVLCQAHGLTPEQVVAIASHDGGKQALETVQRLLPVLCQAHGLTPEQVVAIASNGGGKQALETVQRLLPVLCQAHGLTPEQVVAIASNGGGKQALETVQRLLPVLCQAHGLTPEQVVAIASNNGGKQALETVQRLLPVLCQAHGLTPEQVVAIASNIGGKQALETVQRLLPVLCQAHGLTPEQVVAIASHDGGKQALETVQRLLPVLCQAHGLTPEQVVAIASNIGGKQALETVQRLLPVLCQAHGLTPEQVVAIASNGGGKQALETVQRLLPVLCQAHGLTPEQVVAIASNNGGKQALETVQRLLPVLCQAHGLTPEQVVAIASNIGGKQALEKAS͏**SIVAQLSRPDPALAALTNDHLVALACLGGRPALDAVKKGLPHAPALIKRTNRRIPERTSHRVADHAQVVRVLGFFQCHSHPAQAFDDAMTQFGMSRHGLLQLFRRVGVTELEARSGTLPPASQRWDRILQASGMKRAKPSPTSTQTPDQASLHAFADSLERDLDAPSPMHEGDQTRASSRKRSRSDRAVTGPSAQQSFEVRVPEQRDALHLPLLSWGVKRPRTRIGGLLDPGTPMDADLVASSTVVW͏**GASRELHLDGEDVAMAHADALDDFDLDMLGDGDSPGPGFTPHDSAPYGALDTADFEFEQMFTDALGIDEYGG

1. TALE1

**KGEELFTGVVPILVELDGDVNGHKFSVSGEGEGDATYGKLTLKFICTTGKLPVPWPTLVTTFTYGVQCFSRYPDHMKQHDFFKSAMPEGYVQERTIFFKDDGNYKTRAEVKFEGDTLVNRIELKGIDFKEDGNILGHKLEYNYNSHNVYIMADKQKNGIKVNFKIRHNIEDGSVQLADHYQQNTPIGDGPVLLPDNHYLSTQSALSKDPNEKRDHMVLLEFVTAAGITHGMDELY͏MDPIRSRTPSPARELLSGPQPDGVQPTADRGVSPPAGGPLDGLPARRTMSRTRLPSPPAPSPAFSADSFSDLLRQFDPSLFNTSLFDSLPPFGAHHTEAATGEWDEVQSGLRAADAPPPTMRVAVTAARPPRAKPAPRRRAAQPSDASPAAQVDLRTLGYSQQQQEKIKPKVRSTVAQHHEALVGHGFTHAHIVALSQHPAALGTVAVKYQDMIAALPEATHEAIVGVGKQWSGARALEALLTVAGELRGPPLQLDTGQLLKIANGGGVTAVEAVHAWRNALTGAP͏**NLTPEQVVAIASNGGGKQALETVQRLLPVLCQAHGLTPEQVVAIASNIGGKQALETVQRLLPVLCQAHGLTPEQVVAIASNIGGKQALETVQRLLPVLCQAHGLTPEQVVAIASNIGGKQALETVQRLLPVLCQAHGLTPEQVVAIASNGGGKQALETVQRLLPVLCQAHGLTPEQVVAIASNIGGKQALETVQRLLPVLCQAHGLTPEQVVAIASNIGGKQALETVQRLLPVLCQAHGLTPEQVVAIASNIGGKQALETVQRLLPVLCQAHGLTPEQVVAIASNNGGKQALETVQRLLPVLCQAHGLTPEQVVAIASNIGGKQALETVQRLLPVLCQAHGLTPEQVVAIASNNGGKQALETVQRLLPVLCQAHGLTPEQVVAIASNGGGKQALETVQRLLPVLCQAHGLTPEQVVAIASNGGGKQALETVQRLLPVLCQAHGLTPEQVVAIASNGGGKQALETVQRLLPVLCQAHGLTPEQVVAIASNGGGKQALETVQRLLPVLCQAHGLTPEQVVAIASNIGGKQALETVQRLLPVLCQAHGLTPEQVVAIASNGGGKQALETVQRLLPVLCQAHGLTPEQVVAIASNIGGKQALEKAS͏**SIVAQLSRPDPALAALTNDHLVALACLGGRPALDAVKKGLPHAPALIKRTNRRIPERTSHRVADHAQVVRVLGFFQCHSHPAQAFDDAMTQFGMSRHGLLQLFRRVGVTELEARSGTLPPASQRWDRILQASGMKRAKPSPTSTQTPDQASLHAFADSLERDLDAPSPMHEGDQTRASSRKRSRSDRAVTGPSAQQSFEVRVPEQRDALHLPLLSWGVKRPRTRIGGLLDPGTPMDADLVASSTVVW͏**GASRELHLDGEDVAMAHADALDDFDLDMLGDGDSPGPGFTPHDSAPYGALDTADFEFEQMFTDALGIDEYGG

1. TALE2

**KGEELFTGVVPILVELDGDVNGHKFSVSGEGEGDATYGKLTLKFICTTGKLPVPWPTLVTTFTYGVQCFSRYPDHMKQHDFFKSAMPEGYVQERTIFFKDDGNYKTRAEVKFEGDTLVNRIELKGIDFKEDGNILGHKLEYNYNSHNVYIMADKQKNGIKVNFKIRHNIEDGSVQLADHYQQNTPIGDGPVLLPDNHYLSTQSALSKDPNEKRDHMVLLEFVTAAGITHGMDELY͏MDPIRSRTPSPARELLSGPQPDGVQPTADRGVSPPAGGPLDGLPARRTMSRTRLPSPPAPSPAFSADSFSDLLRQFDPSLFNTSLFDSLPPFGAHHTEAATGEWDEVQSGLRAADAPPPTMRVAVTAARPPRAKPAPRRRAAQPSDASPAAQVDLRTLGYSQQQQEKIKPKVRSTVAQHHEALVGHGFTHAHIVALSQHPAALGTVAVKYQDMIAALPEATHEAIVGVGKQWSGARALEALLTVAGELRGPPLQLDTGQLLKIANGGGVTAVEAVHAWRNALTGAP͏**NLTPEQVVAIASNIGGKQALETVQRLLPVLCQAHGLTPEQVVAIASNNGGKQALETVQRLLPVLCQAHGLTPEQVVAIASNIGGKQALETVQRLLPVLCQAHGLTPEQVVAIASNIGGKQALETVQRLLPVLCQAHGLTPEQVVAIASNIGGKQALETVQRLLPVLCQAHGLTPEQVVAIASHDGGKQALETVQRLLPVLCQAHGLTPEQVVAIASNGGGKQALETVQRLLPVLCQAHGLTPEQVVAIASNIGGKQALETVQRLLPVLCQAHGLTPEQVVAIASHDGGKQALETVQRLLPVLCQAHGLTPEQVVAIASNGGGKQALETVQRLLPVLCQAHGLTPEQVVAIASNIGGKQALETVQRLLPVLCQAHGLTPEQVVAIASNIGGKQALETVQRLLPVLCQAHGLTPEQVVAIASNIGGKQALETVQRLLPVLCQAHGLTPEQVVAIASNIGGKQALETVQRLLPVLCQAHGLTPEQVVAIASNGGGKQALETVQRLLPVLCQAHGLTPEQVVAIASNNGGKQALETVQRLLPVLCQAHGLTPEQVVAIASNGGGKQALETVQRLLPVLCQAHGLTPEQVVAIASNIGGKQALEKAS͏**SIVAQLSRPDPALAALTNDHLVALACLGGRPALDAVKKGLPHAPALIKRTNRRIPERTSHRVADHAQVVRVLGFFQCHSHPAQAFDDAMTQFGMSRHGLLQLFRRVGVTELEARSGTLPPASQRWDRILQASGMKRAKPSPTSTQTPDQASLHAFADSLERDLDAPSPMHEGDQTRASSRKRSRSDRAVTGPSAQQSFEVRVPEQRDALHLPLLSWGVKRPRTRIGGLLDPGTPMDADLVASSTVVW͏**GASRELHLDGEDVAMAHADALDDFDLDMLGDGDSPGPGFTPHDSAPYGALDTADFEFEQMFTDALGIDEYGG

1. TALE3

**KGEELFTGVVPILVELDGDVNGHKFSVSGEGEGDATYGKLTLKFICTTGKLPVPWPTLVTTFTYGVQCFSRYPDHMKQHDFFKSAMPEGYVQERTIFFKDDGNYKTRAEVKFEGDTLVNRIELKGIDFKEDGNILGHKLEYNYNSHNVYIMADKQKNGIKVNFKIRHNIEDGSVQLADHYQQNTPIGDGPVLLPDNHYLSTQSALSKDPNEKRDHMVLLEFVTAAGITHGMDELY͏MDPIRSRTPSPARELLSGPQPDGVQPTADRGVSPPAGGPLDGLPARRTMSRTRLPSPPAPSPAFSADSFSDLLRQFDPSLFNTSLFDSLPPFGAHHTEAATGEWDEVQSGLRAADAPPPTMRVAVTAARPPRAKPAPRRRAAQPSDASPAAQVDLRTLGYSQQQQEKIKPKVRSTVAQHHEALVGHGFTHAHIVALSQHPAALGTVAVKYQDMIAALPEATHEAIVGVGKQWSGARALEALLTVAGELRGPPLQLDTGQLLKIANGGGVTAVEAVHAWRNALTGAP͏**NLTPEQVVAIASNIGGKQALETVQRLLPVLCQAHGLTPEQVVAIASNNGGKQALETVQRLLPVLCQAHGLTPEQVVAIASNNGGKQALETVQRLLPVLCQAHGLTPEQVVAIASNIGGKQALETVQRLLPVLCQAHGLTPEQVVAIASNIGGKQALETVQRLLPVLCQAHGLTPEQVVAIASNIGGKQALETVQRLLPVLCQAHGLTPEQVVAIASNIGGKQALETVQRLLPVLCQAHGLTPEQVVAIASHDGGKQALETVQRLLPVLCQAHGLTPEQVVAIASNGGGKQALETVQRLLPVLCQAHGLTPEQVVAIASNNGGKQALETVQRLLPVLCQAHGLTPEQVVAIASNIGGKQALETVQRLLPVLCQAHGLTPEQVVAIASNIGGKQALETVQRLLPVLCQAHGLTPEQVVAIASNIGGKQALETVQRLLPVLCQAHGLTPEQVVAIASNNGGKQALETVQRLLPVLCQAHGLTPEQVVAIASNNGGKQALETVQRLLPVLCQAHGLTPEQVVAIASNNGGKQALETVQRLLPVLCQAHGLTPEQVVAIASNIGGKQALETVQRLLPVLCQAHGLTPEQVVAIASNNGGKQALEKAS͏**SIVAQLSRPDPALAALTNDHLVALACLGGRPALDAVKKGLPHAPALIKRTNRRIPERTSHRVADHAQVVRVLGFFQCHSHPAQAFDDAMTQFGMSRHGLLQLFRRVGVTELEARSGTLPPASQRWDRILQASGMKRAKPSPTSTQTPDQASLHAFADSLERDLDAPSPMHEGDQTRASSRKRSRSDRAVTGPSAQQSFEVRVPEQRDALHLPLLSWGVKRPRTRIGGLLDPGTPMDADLVASSTVVW͏**GASRELHLDGEDVAMAHADALDDFDLDMLGDGDSPGPGFTPHDSAPYGALDTADFEFEQMFTDALGIDEYGG

1. TALE4

**KGEELFTGVVPILVELDGDVNGHKFSVSGEGEGDATYGKLTLKFICTTGKLPVPWPTLVTTFTYGVQCFSRYPDHMKQHDFFKSAMPEGYVQERTIFFKDDGNYKTRAEVKFEGDTLVNRIELKGIDFKEDGNILGHKLEYNYNSHNVYIMADKQKNGIKVNFKIRHNIEDGSVQLADHYQQNTPIGDGPVLLPDNHYLSTQSALSKDPNEKRDHMVLLEFVTAAGITHGMDELY͏MDPIRSRTPSPARELLSGPQPDGVQPTADRGVSPPAGGPLDGLPARRTMSRTRLPSPPAPSPAFSADSFSDLLRQFDPSLFNTSLFDSLPPFGAHHTEAATGEWDEVQSGLRAADAPPPTMRVAVTAARPPRAKPAPRRRAAQPSDASPAAQVDLRTLGYSQQQQEKIKPKVRSTVAQHHEALVGHGFTHAHIVALSQHPAALGTVAVKYQDMIAALPEATHEAIVGVGKQWSGARALEALLTVAGELRGPPLQLDTGQLLKIANGGGVTAVEAVHAWRNALTGAP͏**NLTPEQVVAIASNNGGKQALETVQRLLPVLCQAHGLTPEQVVAIASNNGGKQALETVQRLLPVLCQAHGLTPEQVVAIASNNGGKQALETVQRLLPVLCQAHGLTPEQVVAIASNIGGKQALETVQRLLPVLCQAHGLTPEQVVAIASNIGGKQALETVQRLLPVLCQAHGLTPEQVVAIASNIGGKQALETVQRLLPVLCQAHGLTPEQVVAIASNGGGKQALETVQRLLPVLCQAHGLTPEQVVAIASNGGGKQALETVQRLLPVLCQAHGLTPEQVVAIASHDGGKQALETVQRLLPVLCQAHGLTPEQVVAIASHDGGKQALETVQRLLPVLCQAHGLTPEQVVAIASNGGGKQALETVQRLLPVLCQAHGLTPEQVVAIASHDGGKQALETVQRLLPVLCQAHGLTPEQVVAIASNGGGKQALETVQRLLPVLCQAHGLTPEQVVAIASNNGGKQALETVQRLLPVLCQAHGLTPEQVVAIASNIGGKQALETVQRLLPVLCQAHGLTPEQVVAIASNIGGKQALETVQRLLPVLCQAHGLTPEQVVAIASNGGGKQALEKAS͏**SIVAQLSRPDPALAALTNDHLVALACLGGRPALDAVKKGLPHAPALIKRTNRRIPERTSHRVADHAQVVRVLGFFQCHSHPAQAFDDAMTQFGMSRHGLLQLFRRVGVTELEARSGTLPPASQRWDRILQASGMKRAKPSPTSTQTPDQASLHAFADSLERDLDAPSPMHEGDQTRASSRKRSRSDRAVTGPSAQQSFEVRVPEQRDALHLPLLSWGVKRPRTRIGGLLDPGTPMDADLVASSTVVW͏**GASRELHLDGEDVAMAHADALDDFDLDMLGDGDSPGPGFTPHDSAPYGALDTADFEFEQMFTDALGIDEYGG

1. TALE5

**KGEELFTGVVPILVELDGDVNGHKFSVSGEGEGDATYGKLTLKFICTTGKLPVPWPTLVTTFTYGVQCFSRYPDHMKQHDFFKSAMPEGYVQERTIFFKDDGNYKTRAEVKFEGDTLVNRIELKGIDFKEDGNILGHKLEYNYNSHNVYIMADKQKNGIKVNFKIRHNIEDGSVQLADHYQQNTPIGDGPVLLPDNHYLSTQSALSKDPNEKRDHMVLLEFVTAAGITHGMDELY͏MDPIRSRTPSPARELLSGPQPDGVQPTADRGVSPPAGGPLDGLPARRTMSRTRLPSPPAPSPAFSADSFSDLLRQFDPSLFNTSLFDSLPPFGAHHTEAATGEWDEVQSGLRAADAPPPTMRVAVTAARPPRAKPAPRRRAAQPSDASPAAQVDLRTLGYSQQQQEKIKPKVRSTVAQHHEALVGHGFTHAHIVALSQHPAALGTVAVKYQDMIAALPEATHEAIVGVGKQWSGARALEALLTVAGELRGPPLQLDTGQLLKIANGGGVTAVEAVHAWRNALTGAP͏**NLTPEQVVAIASHDGGKQALETVQRLLPVLCQAHGLTPEQVVAIASNGGGKQALETVQRLLPVLCQAHGLTPEQVVAIASNNGGKQALETVQRLLPVLCQAHGLTPEQVVAIASNIGGKQALETVQRLLPVLCQAHGLTPEQVVAIASNIGGKQALETVQRLLPVLCQAHGLTPEQVVAIASNGGGKQALETVQRLLPVLCQAHGLTPEQVVAIASNIGGKQALETVQRLLPVLCQAHGLTPEQVVAIASNNGGKQALETVQRLLPVLCQAHGLTPEQVVAIASNIGGKQALETVQRLLPVLCQAHGLTPEQVVAIASNNGGKQALETVQRLLPVLCQAHGLTPEQVVAIASNIGGKQALETVQRLLPVLCQAHGLTPEQVVAIASNNGGKQALETVQRLLPVLCQAHGLTPEQVVAIASNIGGKQALETVQRLLPVLCQAHGLTPEQVVAIASNNGGKQALEKAS͏**SIVAQLSRPDPALAALTNDHLVALACLGGRPALDAVKKGLPHAPALIKRTNRRIPERTSHRVADHAQVVRVLGFFQCHSHPAQAFDDAMTQFGMSRHGLLQLFRRVGVTELEARSGTLPPASQRWDRILQASGMKRAKPSPTSTQTPDQASLHAFADSLERDLDAPSPMHEGDQTRASSRKRSRSDRAVTGPSAQQSFEVRVPEQRDALHLPLLSWGVKRPRTRIGGLLDPGTPMDADLVASSTVVW͏**GASRELHLDGEDVAMAHADALDDFDLDMLGDGDSPGPGFTPHDSAPYGALDTADFEFEQMFTDALGIDEYGG

1. TALE6

**KGEELFTGVVPILVELDGDVNGHKFSVSGEGEGDATYGKLTLKFICTTGKLPVPWPTLVTTFTYGVQCFSRYPDHMKQHDFFKSAMPEGYVQERTIFFKDDGNYKTRAEVKFEGDTLVNRIELKGIDFKEDGNILGHKLEYNYNSHNVYIMADKQKNGIKVNFKIRHNIEDGSVQLADHYQQNTPIGDGPVLLPDNHYLSTQSALSKDPNEKRDHMVLLEFVTAAGITHGMDELY͏MDPIRSRTPSPARELLSGPQPDGVQPTADRGVSPPAGGPLDGLPARRTMSRTRLPSPPAPSPAFSADSFSDLLRQFDPSLFNTSLFDSLPPFGAHHTEAATGEWDEVQSGLRAADAPPPTMRVAVTAARPPRAKPAPRRRAAQPSDASPAAQVDLRTLGYSQQQQEKIKPKVRSTVAQHHEALVGHGFTHAHIVALSQHPAALGTVAVKYQDMIAALPEATHEAIVGVGKQWSGARALEALLTVAGELRGPPLQLDTGQLLKIANGGGVTAVEAVHAWRNALTGAP͏**NLTPEQVVAIASHDGGKQALETVQRLLPVLCQAHGLTPEQVVAIASNGGGKQALETVQRLLPVLCQAHGLTPEQVVAIASHDGGKQALETVQRLLPVLCQAHGLTPEQVVAIASNIGGKQALETVQRLLPVLCQAHGLTPEQVVAIASNGGGKQALETVQRLLPVLCQAHGLTPEQVVAIASNIGGKQALETVQRLLPVLCQAHGLTPEQVVAIASNGGGKQALETVQRLLPVLCQAHGLTPEQVVAIASNIGGKQALETVQRLLPVLCQAHGLTPEQVVAIASNIGGKQALETVQRLLPVLCQAHGLTPEQVVAIASNIGGKQALETVQRLLPVLCQAHGLTPEQVVAIASNGGGKQALETVQRLLPVLCQAHGLTPEQVVAIASNIGGKQALETVQRLLPVLCQAHGLTPEQVVAIASNNGGKQALETVQRLLPVLCQAHGLTPEQVVAIASNNGGKQALETVQRLLPVLCQAHGLTPEQVVAIASHDGGKQALETVQRLLPVLCQAHGLTPEQVVAIASHDGGKQALETVQRLLPVLCQAHGLTPEQVVAIASNIGGKQALETVQRLLPVLCQAHGLTPEQVVAIASNGGGKQALEKAS͏**SIVAQLSRPDPALAALTNDHLVALACLGGRPALDAVKKGLPHAPALIKRTNRRIPERTSHRVADHAQVVRVLGFFQCHSHPAQAFDDAMTQFGMSRHGLLQLFRRVGVTELEARSGTLPPASQRWDRILQASGMKRAKPSPTSTQTPDQASLHAFADSLERDLDAPSPMHEGDQTRASSRKRSRSDRAVTGPSAQQSFEVRVPEQRDALHLPLLSWGVKRPRTRIGGLLDPGTPMDADLVASSTVVW͏**GASRELHLDGEDVAMAHADALDDFDLDMLGDGDSPGPGFTPHDSAPYGALDTADFEFEQMFTDALGIDEYGG

**Table S2**: DNA oligonucleotides applied for qRT-PCR

| target |  | sequence |
| --- | --- | --- |
| IFNβ | fw | AGG AGG ACG CCG CAT TGA CC |
|  | rev | GAT GAT AGA CAT TAG CCA GGA GGT TCT CAA C |
| HCV-JFH | fw | TCG ACG TTG TTA CAA GGT CTC C |
|  | rev | CTG CCA GTT GGA GCA TGC |
| GAPDH | fw | AGC CTC AAG ATC ATC AGC AAT GC |
|  | rev | ATG GCA TGG ACT GTG GTC ATG |
| OAS1 | fw | GAA ACT TGG GTG GTG GAG AC |
|  | rev | CTT TCA GCC AGC AGA ATC CA |
| OAS2 | fw | GAA TGG TTA TCC TCT CCC TGC |
|  | rev | ATC CTA GCT CCA CAA CTT CCT G |
| Mx1 | fw | GGT CAG AGA GAA GGA GCT GG |
|  | rev | CTC CTG GTG ATA GGG CAT CA |
| IRF7 | fw | AGA GCC TGG TCC TGG TGA AG |
|  | rev | CAT AGA GGC TGT TGG CGC TG |
| ADAR1 | fw | TGT GGA TGG GCC ACG GAA TG |
|  | rev | GCA GCT TTC TTG GCC TCA CCA TAG |
